# Elastography of the bone-implant interface

**DOI:** 10.1038/s41598-019-50665-4

**Published:** 2019-10-02

**Authors:** Yoann Hériveaux, Vu-Hieu Nguyen, Didier Geiger, Guillaume Haïat

**Affiliations:** 1 CNRS, Laboratoire Modélisation et Simulation Multi Echelle, MSME UMR 8208 CNRS, 61 avenue du Général de Gaulle, 94010 Créteil Cedex, France; 2 Université Paris-Est, Laboratoire Modélisation et Simulation Multi Echelle, MSME UMR 8208 CNRS, 61 avenue du Général de Gaulle, 94010 Créteil Cedex, France

**Keywords:** Imaging techniques, Biomedical engineering, Preclinical research

## Abstract

The stress distribution around endosseous implants is an important determinant of the surgical success. However, no method developed so far to determine the implant stability is sensitive to the loading conditions of the bone-implant interface (BII). The objective of this study is to investigate whether a quantitative ultrasound (QUS) technique may be used to retrieve information on compressive stresses applied to the BII. An acousto-mechanical device was conceived to compress 18 trabecular bovine bone samples onto coin-shaped implants and to measure the ultrasonic response of the BII during compression. The biomechanical behavior of the trabecular bone samples was modeled as Neo-Hookean. The reflection coefficient of the BII was shown to decrease as a function of the stress during the elastic compression of the trabecular bone samples and during the collapse of the trabecular network, with an average slope of −4.82 GPa^−1^. The results may be explained by an increase of the bone-implant contact ratio and by changes of bone structure occurring during compression. The sensitivity of the QUS response of the BII to compressive stresses opens new paths in the elaboration of patient specific decision support systems allowing surgeons to assess implant stability that should be developed in the future.

## Introduction

Endosseous cementless titanium implants are now widely used in orthopedic, dental and maxillofacial surgeries^1,2^. However, despite a routine clinical use, osseointegration failures still occur and may have dramatic consequences. The implant surgical success is directly determined by the evolution of the biomechanical properties of the bone-implant interface (BII)^3–5^.

During surgery, endosseous implants are inserted in a slightly undersized bone cavity formed by drilling or cutting, leading to a pre-stressed state of the bone-implant system referred to as primary implant stability. A compromise should be found between (i) insufficient primary stability leading to excessive interfacial micromotion following surgery^6–8^, which may imply implant migration^9^ and failure and (ii) excessive stresses at the BII, which may lead to bone necrosis^10,11^.

During healing, osseointegration phenomena, corresponding to an apposition of bone tissue around the implant surface, are stimulated by “low level” stresses applied to the BII^12^, but excessive level of stresses may damage the consolidating BII and lead to implant failure.

As a consequence, the stress distribution around the implant during and after surgery is an important determinant for the implant success^13^, but it remains difficult to be assessed experimentally. X-ray based techniques^14^ and magnetic resonance imaging^15^ cannot be used to assess the level of stress at the BII due to diffraction phenomena related to the presence of metal. Therefore, biomechanical methods are needed.

An interesting approach to assess the level of stress at the BII consists in employing finite element analysis (FEA). For example, stress and strain fields have been predicted around the BII in the context of dental^16,17^ and orthopedic implants applications^18^. The results showed that stresses in the range of 0–10 MPa could be obtained at the BII, depending on the physiological boundary conditions. However, despite the progresses realized in computational analyses, it remains difficult to assess in a patient specific manner the loading conditions at the BII due to the complexity of the implant geometry and of the bone material properties.

Different biomechanical techniques have been developed to assess implant stability. For example, percussion test methods based on the measurement of the contact duration between the implant and the impacting device have been developed in the context of dental^19^ and orthopedic surgery^20,21^. The most commonly used biomechanical technique is the resonance frequency analysis (RFA)^22^, which consists in measuring the first bending resonance frequency of a small rod attached to the implant^23^. However, to the best of our knowledge, none of the aforementioned techniques is capable of retrieving the loading conditions at the BII.

Interestingly, quantitative ultrasound (QUS) has emerged as a promising method to retrieve information on the BII. QUS has the advantage to be non-invasive, non-radiating and relatively cheap. The principle of QUS measurements lies on the dependence of the ultrasonic propagation at the BII on the bone-implant contact ratio (BIC) and on the bone mechanical properties. A combined increase of the BIC and of the periprosthetic bone Young’s modulus^24^ and mass density^25,26^ occurs during healing and leads to a decrease of the reflection coefficient at the BII due to a decrease of the gap of acoustical properties, a phenomenon that has been evidenced both experimentally^27^ and *in silico*^28,29^. Based on these results and on a preliminary study^30^, a QUS device has been developed by our group to assess dental implant stability. Preliminary validation was performed *ex vivo* using cylindrical implants^31^ and dental implants inserted in a bone substitute biomaterial^32^ and in bovine bone tissue^33^. This QUS device, which consists in screwing a 10 MHz monoelement transducer into dental implants, was then validated *in vivo*^34^ and *in silico*^35–37^. More recent studies showed that the reproducibility and the sensitivity of the QUS device were significantly better compared to the results obtained *in vitro*^38^ and *in vivo*^39^ with resonance frequency analysis. In particular, the results indicated that the QUS device was significantly more sensitive to the final drill diameter compared to the resonance frequency analysis method^38^. However, it remains impossible to assess the influence of the mechanical stresses applied to the BII on its ultrasonic response in a controlled manner.

The aim of the present work is to determine whether a QUS technique may be used to assess the effect of compressive stresses on the ultrasonic response of the BII. To do so, trabecular bovine bone samples were progressively compressed onto coin-shaped implants. The ultrasonic response of the BII was measured throughout the compression stage in order to retrieve the reflection coefficient of the compressed BII.

The first section describes the experiments and their analysis consisting of compressing bone tissue on an implant and of measuring the ultrasonic response of the BII. The second section presents the main results of the study, in particular regarding the correlation found between (i) stresses applied to the BII and its ultrasonic response and (ii) bone properties and the ultrasonic response of the BII. Finally, the results are discussed in the last section.

## Material and Methods

### Bone samples and implant

All bovine bone samples were obtained from the butcher shop so the experiments were not carried out on live animals. Eighteen trabecular bone samples were cut from three bovine femoral heads. Each cubic sample had dimensions of around 14 × 14 × 14 mm that were measured with a caliper. Each sample was also weighed and its apparent density *ρ* was determined. A mirror-polished Ti-Al6-V4 titanium alloy coin-shaped implant (20 mm dimeter and 5 mm thickness) was used throughout the study.

### Compression device

**Figure 1:**
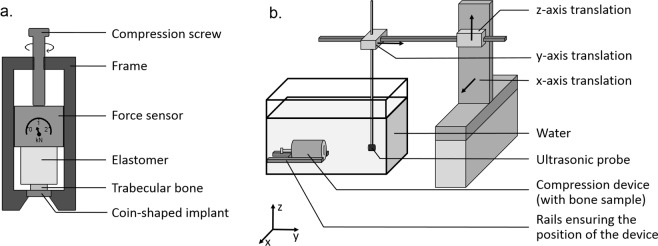
Schematic illustrations of the devices used (**a**) to compress the bone sample and measure the applied stress and (**b**) to realized ultrasonic measurements.

Figure 1a shows a schematic description of the device used to compress the bone sample onto the implant surface and to measure the stress applied to the BII. The device was composed of the coin-shaped implant, the bone sample, an elastomer cylinder made of polyurethane and a force sensor stacked inside a rigid cylindrical frame. The elastomer cylinder (35 cm long and 30 cm diameter at rest) acted as an elastic spring aiming at applying forces to the bone sample and to the BII. The force sensor measured the force *F* applied to the elastomer, which allowed to deduce the stress *σ* at the BII. Friction phenomena between the sensor and the frame were minimized using lubrication. The compression was realized by tightening a screw with a thread pitch of 1.5 mm positioned on the opposite side of the device compared to the implant, allowing to control the displacement at one end of the elastomer.

### Ultrasonic measurements

The ultrasonic probe consisted in a broadband focused immersed transducer (CMF-25; Sonaxis, Besançon, France) with a center frequency equal to 15 MHz, a diameter of 6 mm and a focal length of 40 mm, which led to a beam width at the focus approximately equal to 0.5 mm in water. The transducer acted as an emitter-receiver. The supporting electronics comprised a pulse-receiver amplifier and an A/D conversion card of 100-MHz sampling frequency.

As shown in Fig. 1b, the ultrasonic measurements were performed by immersing the device described in Fig. 1a in a container filled with water at room temperature. The compression device was positioned in the container so that (i) the BII was located approximately at the focus of the transducer and (ii) the normal of the implant surface and of the axis of the transducer coincided with the *y*-direction, with a maximum parallelism error of 1°. The displacement of the probe for 2-D imaging was controlled using two translation stages moving in the *x* and *z* directions and fixed to a rigid frame (see Fig. 1b). A spatial acquisition square window of 8 × 8 mm centered on the axis of the coin-shaped implant was considered with a displacement step equal to 1 mm. A total number of 64 radiofrequency signals were recorded for each ultrasonic measurement and *i* denotes the number of the signal recorded. The reproducibility of the ultrasonic measurements was assessed by repositioning a given bone sample and repeating the procedure six times with the same force (50N) applied to the BII.

For each bone sample, the first ultrasonic measurement (corresponding to the 2-D scan described above) was performed with a force *F* equal to 50N applied to the BII. Then, the compression screw was tightened by 2π rad, which corresponds to a displacement of 1.5 mm, and the ultrasonic measurements were reproduced. The compression screw was tightened by 2π rad six more times in order to realize ultrasonic measurements for six increasing values of the force applied to the BII. Then, the compression screw was tightened by 4π rad and the ultrasonic measurements were reproduced. This process of tightening the compression screw by 4π rad and reproducing the ultrasonic measurements was carried out until the force reached a value of 2 kN. The upper limit equal to 2 kN for the force was considered because it corresponds to a compressive stress *σ* equal to around 10.2 MPa on the BII, which approximately corresponds to the maximum stress simulated at the BII using FEA^16–18^. An homogeneous stress distribution at the BII was assumed. A total number of *N* loading steps (which corresponds to *N* values of the force *F*) was considered and *j *∈ {1, *N*} corresponds to the running number of the loading step.

The force applied to the BII was measured at the beginning and at the end of each ultrasonic measurement (which lasted around 500 seconds) in order to account for effects related to the stress relaxation of the system (see below).

### Analysis of the compressive stress and strain applied to bone samples

Due to stress relaxation effects, the compressive stress applied to the BII decreased during the 500 seconds corresponding to the duration of the ultrasonic measurement. The values of the compressive stress applied to the BII obtained at the beginning and at the end of the ultrasonic measurement corresponding to loading step #*j* were noted *σ*^+^(*j*) and *σ*^*−*^(*j*) respectively. The average and standard deviation value of *σ*^+^(*j*) and *σ*^*−*^(*j*) were denoted *σ*^*m*^(*j*) and *σ*^*sd*^(*j*), respectively.

The deformations of the force sensor, of the coin-shaped implant and of the frame were neglected compared to the deformation of the elastomer and of the bone sample, leading to:

1 $$\varepsilon (j)=\frac{\Delta {h}_{T}-\Delta {h}_{E}}{{h}_{b}},$$

where *ε*(*j*) corresponds to the strain of the bone sample, Δ*h*_*T*_ is the displacement imposed by the compression screw (see Fig. 1a), Δ*h*_*E*_ is the variation of length of the elastomer and *h*_*b*_ is the initial length of the bone sample at rest.

The error realized on Δ*h*_*T*_ was given by the uncertainty on the rotation of the screw, which was of the order of 5°, leading to an error on Δ*h*_*T*_ of 0.02 mm.

A compression test of the elastomer was carried out without any bone sample and reproduced three times in order to assess its constitutive law. The following linear elastic macroscopic law was found:

2 $$\Delta {h}_{E}=7,{35.10}^{-6}\,F,$$

Uncertainties on Δ*h*_*E*_ were directly linked to uncertainties on *σ*(*j*) since *F* varies as a function of time between the beginning and the end of the ultrasonic acquisition. The uncertainty on *ε*(*j*) was defined as the sum of the contributions of the uncertainties on Δ*h*_*T*_ and on Δ*h*_*E*_ by the relation:

3 $${\varepsilon }^{sd}(j)=\frac{0,\,{02.10}^{-3}+7,\,{35.10}^{-6}\,S\,({\sigma }^{+}(j)-{\sigma }^{-}(j))}{{h}_{b}},$$

where *S* is the surface of the bone sample in contact with the implant.

### Bone constitutive behavior

The constitutive law of the trabecular bone samples was considered to be Neo-Hookean, following previous studies^40–42^. Such behavior consists in three regimes. *σ* first varies linearly as a function of *ε*, then reaches a nearly constant value, and eventually increases again as a function of *ε*. Here, we assumed a linear dependence of *σ* as a function of *ε* for this last regime, so that the relation between *σ* and *ε* could be interpolated by:

4 $$\tilde{\sigma }(\varepsilon )=\{\begin{array}{ll}{\sigma }_{1}\,\frac{\varepsilon }{{\varepsilon }_{i}} & if\,\varepsilon \le {\varepsilon }_{i}\,\\ {\sigma }_{1} & if\,{\varepsilon }_{i}\le \varepsilon \le {\varepsilon }_{f}\\ {\sigma }_{1}+B.(\varepsilon -{\varepsilon }_{f}) & if\,\varepsilon \ge {\varepsilon }_{f}\end{array}$$

where *ε*_*i*_ and *ε*_*f*_ are the strain values delimiting the different regimes, *σ*_1_ is the stress value at the plateau and *B* is the slope of the curve representing the variation of *σ* as a function of *ε* during the final regime.

Based on the experimental results, a cost function ${e}_{\sigma }(B,\,{\sigma }_{1},{\varepsilon }_{i},{\varepsilon }_{f})$ was defined in order to assess the difference between the experimental measurements and values given by Eq. (4) following:

5 $${e}_{\sigma }(B,\,{\sigma }_{1},{\varepsilon }_{i},{\varepsilon }_{f})=\mathop{\sum }\limits_{j=1}^{N}\,\frac{|\sigma (j)-\tilde{\sigma }(\varepsilon (j))|}{N},$$

An optimization procedure based on a conjugate gradient method was carried out in order to determine the optimal values of the parameters ($B,\,{\sigma }_{1},{\varepsilon }_{i},{\varepsilon }_{f}$) minimizing the cost function *e*_*σ*_ for each bone sample.

### Data analysis of ultrasonic measurements

The same signal processing as the one used in Mathieu *et al*.^27^ was applied to the signals obtained using the ultrasonic device. Briefly, the envelope of each radiofrequency (rf) signal was determined by computing the modulus of its Hilbert’s transform. For each rf signal #*i*, three echoes were considered corresponding to the reflection of the ultrasound wave on (i) the water-implant interface, (ii) the BII and (iii) the BII, the implant-water interface and again the BII. The maximum amplitude of the envelope of the echo #1 (respectively of echoes #2 and #3) corresponding to the rf signal #*i* and to the loading step #*j* was denoted *A*_*i,1*_*(j)* (respectively *A*_*i*,2_*(j)* and *A*_*i*,3_*(j)*). The time window was centered on the time of the maximum of the corresponding echo and had a total length equal to the signal duration (0.9 µs).

The following analysis was carried out for all 18 bone samples. For each loading step *#j*, the average value of the ratio *R*_2_*(j)* (respectively *R*_3_*(j)*) of the amplitudes of echo #2 (respectively echo #3) and echo #1 was calculated over the 8 mm × 8 mm window following:

6 $${R}_{2}(j)=\frac{1}{64}\mathop{\sum }\limits_{i=1}^{64}\,\frac{{A}_{i,2}(j)}{{A}_{i,1}(j)}$$

7 $${R}_{3}(j)=\frac{1}{64}\mathop{\sum }\limits_{i=1}^{64}\,\frac{{A}_{i,3}(j)}{{A}_{i,1}(j)}$$

The reproducibility of the ultrasonic measurements was assessed by determining the values obtained for *R*_*k*_ (*k  *∈ {1, 2}) for each of the six measurements realized with the same sample with repositioning. The reproducibility *a*_*k*_ of the measurement of *R*_*k*_ was defined as the standard deviation obtained for the six corresponding values of *R*_*k*_.

The variation of *R*_*k*_ as a function of *σ* was interpolated by a continuous function linear by pieces of *σ* following:

8 $$\tilde{{R}_{k}}(\sigma )=\{\begin{array}{ll}{\lambda }_{i,k}.\sigma +{R}_{0,k} & if\,\sigma \le {\sigma }_{k}\\ {\lambda }_{f,k}.(\sigma -{\sigma }_{k})+{\lambda }_{i,k}.{\sigma }_{k}+{R}_{0,k} & if\,\sigma \ge {\sigma }_{k}\end{array}$$

where *σ*_*k*_ corresponds to the stress value at which the change of the slope occurs. *λ*_*i*,*k*_(respectively *λ*_*f*,*k*_) represents the initial (respectively final) slope of the linear interpolation of $\tilde{{R}_{k}}(\sigma )$, and *R*_0,*k*_ represents the initial ratio when *σ* = 0. Again, based on the experimental results, a cost function *e*_*R,k*_ *(λ*_*i,k*_, *λ*_*f,k*_*,R*_*0,k*_*,σ*_*k*_*)* was defined in order to assess the difference between the experimental measurements and values obtained with Eq. (8) following:

9 $${e}_{R,k}({\lambda }_{i,k},{\lambda }_{f,k},{R}_{0,k},{\sigma }_{k})=\mathop{\sum }\limits_{j=1}^{N}\,\frac{|{R}_{k}(j)-\tilde{{R}_{k}}(\sigma (j))|}{N}$$

An optimization procedure based on a conjugate gradient method was carried out in order to determine the optimal values of the parameters (*λ*_*i*,*k*_, *λ*_*f*,*k*_, *R*_0,*k*_, *σ*_*k*_) minimizing the cost function *e*_*R*,*k*_ for each trabecular bone sample.

Eventually, a simple method was derived in order to assess the error made on the estimation of the stress based on the ultrasonic measurement for *σ* < *σ*_*k*_. Assuming a linear variation of *R*_*k*_ as a function of *σ*, the precision on the estimation of the stress Δ*σ*_*k*_ at the BII was defined by the relation:

10 $$\Delta {\sigma }_{k}={a}_{k}.{\lambda }_{i,k}$$

## Results

### Neo-Hookean behavior of bone

**Figure 2:**
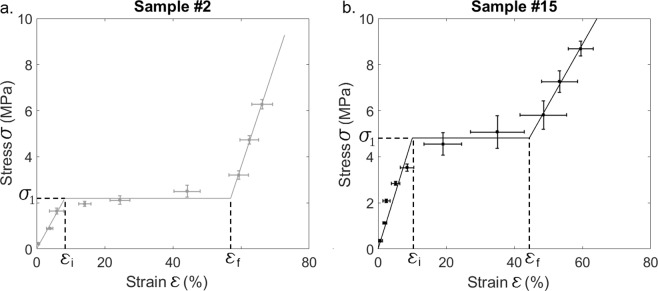
Variation of the stress applied to the bone-implant interface as a function of the strain obtained for bone samples #2 (**a**) and #15 (**b**). Parameters σ_1_, *ε*_*i*_ and *ε*_*f*_ corresponding to the Neo-Hookean behavior of bone tissue are indicated. The solid lines represent the optimal functions $\tilde{\sigma }$ corresponding to each bone sample.

Figure 2 shows the variation of *σ*(*ε*) for bone samples #2 and #15 corresponding to apparent densities of 0.475 g/cm^3^ and 0.736 g/cm^3^ respectively. The results illustrate the three regimes of the Neo-Hookean behavior of trabecular bone (*ε* < *ε*_*i*_, *ε*_*i*_ < *ε* < *ε*_*f*_ and *ε* > *ε*_*f*_) described in the Material and Methods section (see Eq. 4). The error bars relative to *σ* and to ε correspond to *σ*^*sd*^ and *ε*^*sd*^, and increase with stress relaxation effects, in particular for *σ* ≥ *σ*_1_.

Table 1 shows the average, minimum and maximum values of *σ*_1_, *ε*_*i*_, *ρ* and *ε*_*f*_ and their standard variations for the 18 bone samples.

**Table 1:** Mean, minimum, maximum values and standard variation of parameters *σ*_1_, *ε*_*i*_ and *ε*_*f*_ describing the neo-hookean behavior of bone and of the apparent density *ρ* of samples.

Parameter	Mean (+/−SD)	Min	Max
σ_1_ (MPa)	4.34 +/− 1.37	2.19	6.58
ε_i_	8.8% +/− 3.1%	4.60%	17.50%
ε_f_	46.3% +/− 14.1%	18.90%	68.30%
*ρ* (g/cm^3^)	0.661 +/− 0.130	0.451	0.929

### Ultrasonic response of the BII

**Figure 3:**
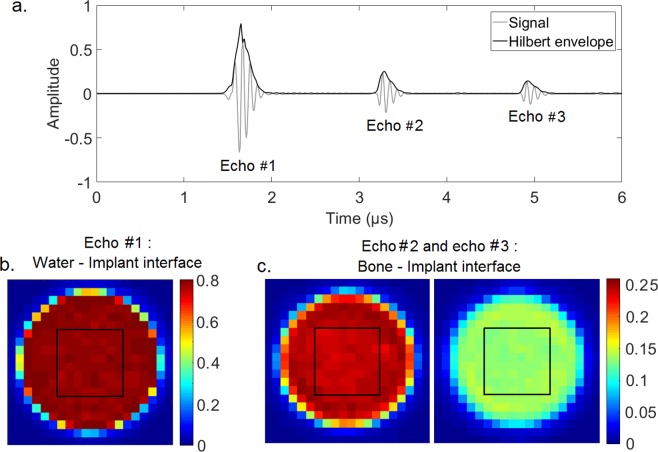
(**a**) Representation of a radiofrequency signal and of its envelop corresponding to an echo obtained with the coin-shaped implant. Echo #1 (respectively #2) corresponds to the echo of the water-implant interface (respectively the BII). Echo #3 corresponds to the rebound of the ultrasonic wave on the BII, on the implant-water interface and on the BII. (**b**) 2-D C-scan corresponding to the maximum amplitude of echo#1 in a 20 × 20 mm spatial window. (**c**) 2-D C-scans corresponding to the maximum amplitude of echo#2 and #3 in a 20 × 20 mm spatial window. The 8 × 8 mm measurement windows realized to assess R_2_ and R_3_ are represented by black squares in Fig. 3b,c.

Figure 3a shows the three echoes described in the last part of the Material and Methods section. Figure 3b,c show the 2-D images obtained by plotting the maximum amplitudes *A*_*i,1*_, *A*_*i*,2_ and *A*_*i*,3_ of the rf signals on a 20 × 20 mm window corresponding to the position of the transducer relatively to the sample in the *x-z* directions. The chosen window size allows to determine the position of the coin shaped implant in the plan perpendicular to the transducer axis. However, in the rest of the study, 2-D scans were only performed on 8 × 8 mm windows centered on the axis of the coin-shaped implant because (i) it allowed to avoid edge effects and (ii) it reduced the time required to obtain each ultrasonic image.

**Figure 4:**
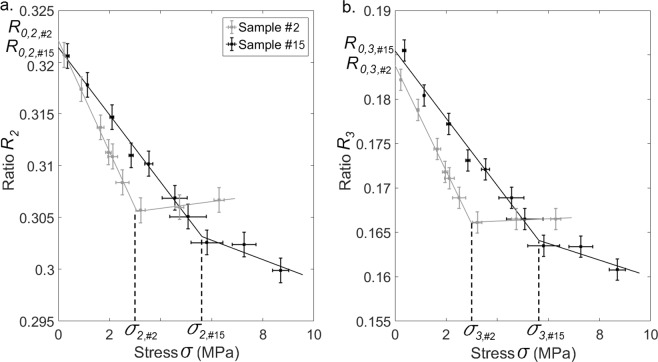
Variation of (**a**) R_2_ and (**b**) R_3_ as a function of the stress σ at the BII for bone samples #2 and #15. Parameters *σ*_*k*_and *R*_0,*k*_ (k ∈ [2, 3]) are indicated, subscript _#m_ corresponding to sample #m. Solid lines represent the optimal functions ${\tilde{R}}_{2}$ and ${\tilde{R}}_{3}$ corresponding to each bone sample. σ_2,#2_ and σ_2,#15_ correspond to the value of σ_2_ obtained for the sample #2 and #15, respectively. σ_3,#2_ and σ_3,#15_ correspond to the value of σ_3_ obtained for the sample #2 and #15, respectively.

The black (respectively grey) line in Fig. 4 shows the variation of *R*_2_ (respectively *R*_*3*_) as a function of the stress *σ* applied to the BII for bone samples #2 and #15. The solid lines represent the optimal functions ${\tilde{R}}_{2}$ (respectively ${\tilde{R}}_{3}$) and indicate a change of slope occurring for *σ*_2_ (respectively *σ*_3_). The reproducibility of the ultrasonic measurements was equal to *a*_*2*_ = 1.2.10^−3^ for *R*_*2*_ and of *a*_3_ = 8.0.10^−4^ for *R*_*3*_.

Table 2 shows the values obtained for the parameters (*λ*_*i*,*k*_, *λ*_*f*,*k*_, *R*_0,*k*_, *σ*_*k*_) describing the variation of *R*_2_ and *R*_3_ as a function of *σ*. For *k* ∈ {*1*, 2}, the values of *λ*_*i,k*_ are negative for all bone samples, which indicates that *R*_*2*_ and *R*_3_ always decrease as a function of *σ* for *σ* < *σ*_*k*_. However, the values of *λ*_*f,k*_ may be positive or negative depending on the sample, which indicates that no specific behavior was obtained for *R*_2_ and *R*_3_, which weakly depend on *σ* for *σ*  > *σ*_*k*_.

**Table 2:** Mean, minimum and maximum values of the parameters describing the evolution of the ultrasonic ratios.

	Parameter	Mean (+/−SD)	Min	Max
**Ratio 2**	R_0,2_	0.323 +/− 0.009	0.307	0.34
	σ_2_ (MPa)	5.05 +/− 1.39	3.05	7.8
	λ_i,2_ (GPa^-1^)	−4.82 +/− 1.95	−8.65	−1.24
	λ_f,2_ (GPa^−1^)	−0.51 +/− 1.29	−2.35	1.84
	Δσ_2_ (MPa)	0.633 +/− 0.424	0.277	1.937
**Ratio 3**	R_0,3_	0.186 +/− 0.009	0.171	0.203
	σ_3_ (MPa)	4.90 +/− 1.41	2.97	7.8
	λ_i,3_ (GPa^−1^)	−5.46 +/− 1.84	−9.46	−2.28
	λ_f,3_ (GPa^−1^)	−0.75 +/− 1.17	−2.54	1.5
	Δσ_3_ (MPa)	0.334 +/− 0.141	0.169	0.701

Table 2 also shows the values of Δ*σ*_*k*_. For a given bone sample, Δ*σ*_3_ is always lower than Δ*σ*_2_, which indicates that considering echo #3 gives a better sensitivity of the ultrasonic response of the BII on variations of stresses compared to considering echo #2. This result may be explained by the fact that echo #3 results from two successive reflections of the ultrasonic wave on the BII whereas echo #2 results from a single reflection on the BII.

**Figure 5:**
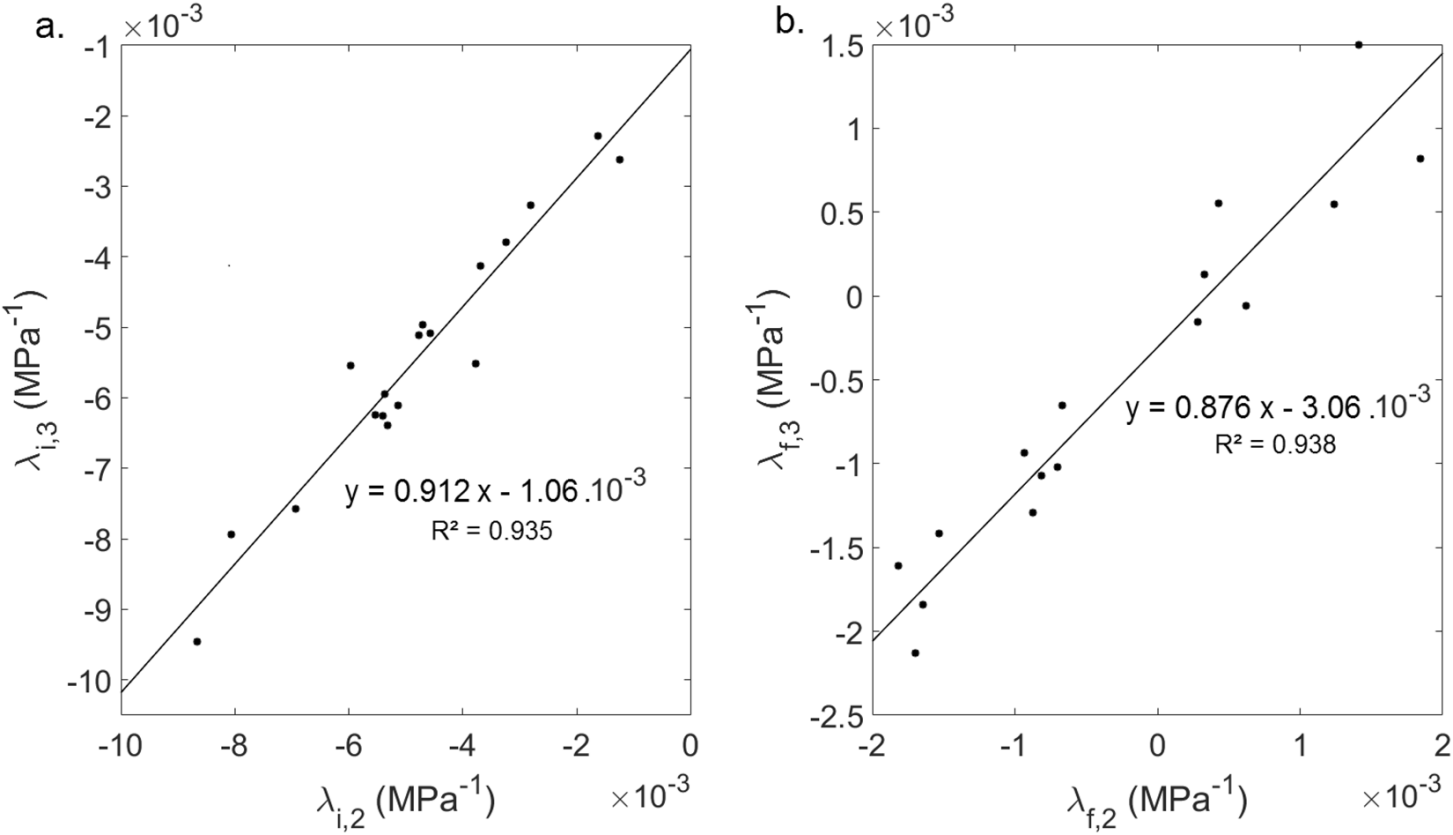
Variations of (**a**) *λ*_*i,3*_ as a function of *λ*_*i,2*_ and (**b**) *λ*_*f,3*_ as a function of *λ*_*f,2*_. The solid lines correspond to linear regression analysis.

Figure 5(a) (respectively 5(b)) shows the variation of *λ*_*i,3*_ as a function of *λ*_*i*,2_ (respectively *λ*_*i,3*_ as a function of *λ*_*i,2*_). The results show that there is a significant correlation between (i) *λ*_*i,3*_ and *λ*_*i,2*_ and (ii) *λ*_*f,2*_ and *λ*_*f,3*_, which indicates that results obtained for echo #2 are consistent with results obtained for echo #3.

### Relation between mechanical and ultrasonic measurements

**Figure 6:**
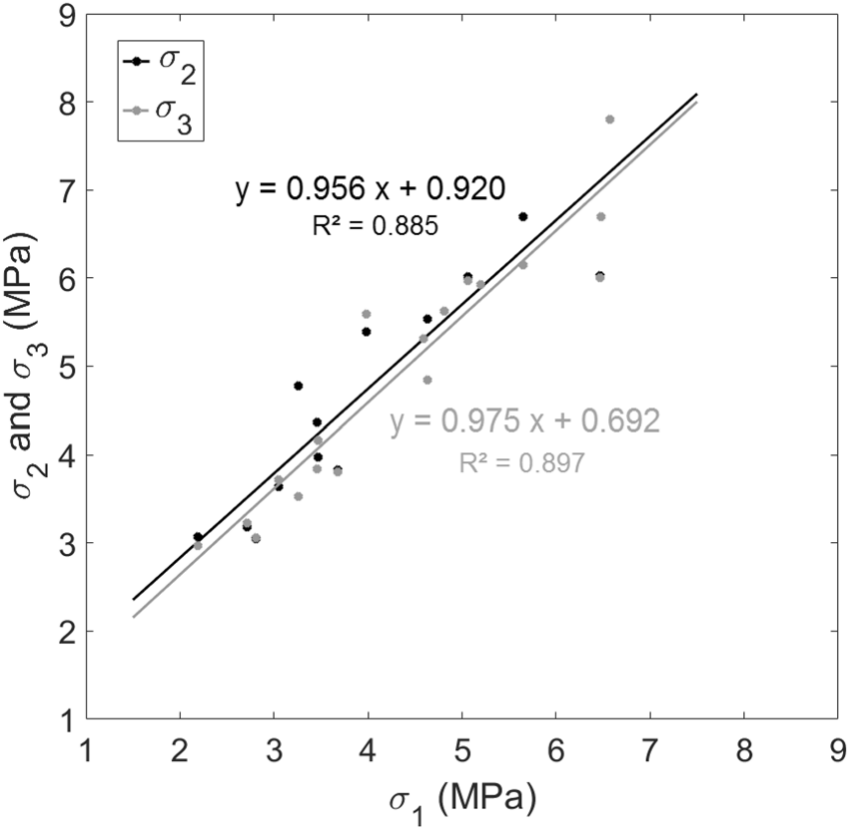
Variation of σ_2_ and σ_3_ (see Fig. 4) as a function of σ_1_ (see Fig. 2). The solid lines correspond to linear regression analysis.

Figure 6 shows the variation of *σ*_*2*_ and *σ*_3_ (derived from the ultrasonic measurements, see Eq. (8)) as a function of *σ*_1_ (derived from the mechanical measurements, see Eq. (4)). A significant correlation is obtained between *σ*_1_ and *σ*_2_ and between *σ*_1_ and *σ*_3_, which indicates that the results obtained from ultrasonic measurements are directly related to the mechanical behavior of the BII. Moreover, the results shown in Fig. 6 indicate that *σ*_2_ > *σ*_1_ and *σ*_3_* > σ*_1_ for 17 out of 18 samples.

**Figure 7:**
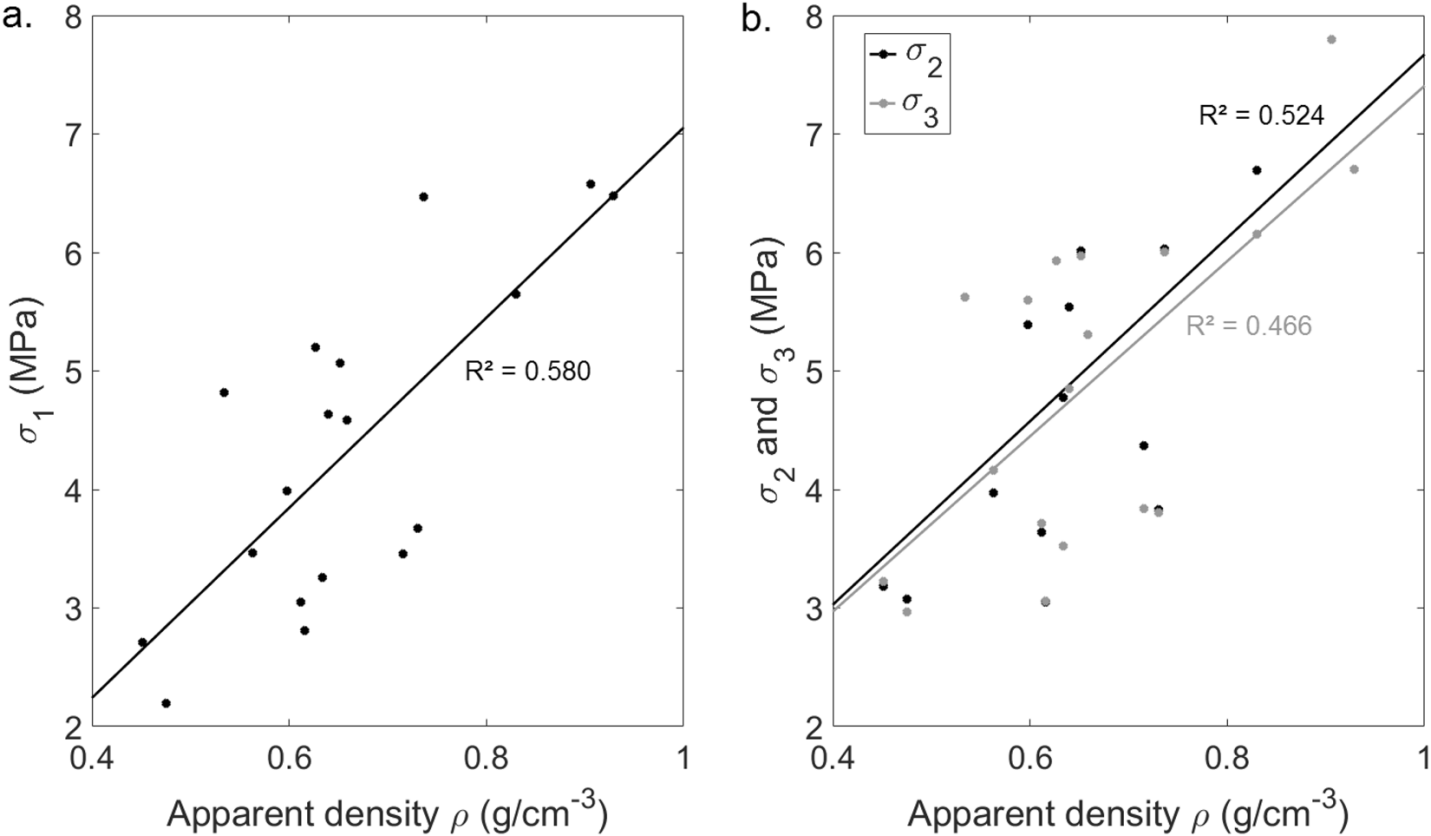
Variation of σ_1_ (**a**) and of σ_2_ and σ_3_ (**b**) as a function of the apparent density of the bone samples ρ. The solid lines correspond to linear regression analysis.

Figure 7 shows the relationship between (i) *σ*_*1*_, *σ*_2_ and *σ*_3_ and (ii) the apparent density *ρ* of the bone samples. The results show that *σ*_*1*_, *σ*_2_ and *σ*_3_ tend to increase as a function of *ρ*.

## Discussion

The originality of the present study is to evidence the dependence of the ultrasonic response of the BII on the compressive stress applied to this same interface. To do so, a dedicated set up coupling mechanical testing and ultrasound measurements was conceived in order to work under standardized conditions. The results showed a strong elastoacoustic coupling for the behavior of the BII.

Previous studies have shown both numerically^28,35,36^ and experimentally^24,34,38^ that the amplitude of the ultrasonic response of the BII decreases as a function of healing time. It was explained by a combined effect of the increase of the BIC ratio and of the periprosthetic bone material properties, leading to a decrease of the gap of acoustical properties at the BII. These results concerning the effect of healing time are in agreement with the results obtained herein because an increase of the compressive stresses at the BII is likely to lead to (i) an increase of the BIC due to local deformation of bone tissue near the BII and (ii) an increase of the bone material properties due to compression.

Moreover, in a recent study^38^, the amplitude of the ultrasonic response of a dental implant was shown to decrease when the diameter of the hole where the implant was inserted decreased, which is also consistent with the results obtained herein under standardized conditions.

The results obtained in the present study could be compared more quantitatively with the results obtained in experimental^27^ and numerical^28^ studies focusing on echo #2 of the ultrasonic response of the BII, allowing a comparison with the results found for the variation of *R*_2_ in the present work. First, Mathieu *et al*.^27^ showed that the reflection coefficient decreased by 7.8% when the BIC increased from around 27% to around 69%, which corresponds to an increase of the BIC equal to 42%. In the present study, bovine trabecular bone, which was taken from the femoral head, had a porosity around 50%^43^. The compression applied by the set up was likely to increase the BIC from around 50% up to a maximum of 100% for very strong compression stresses and was associated to a decrease of *R*_2_ equal to 7.2% from σ = 0.25 MPa to σ = *σ*_2_, which is in good agreement with the experimental results of Mathieu *et al*.^27^. Second, Hériveaux *et al*.^28^ found that the reflection coefficient obtained for implants with a low roughness amplitude equal to 5 µm decreased by 9.3% when the BIC varied from 50% to 100%, which is also in quantitative agreement with results obtained herein.

The results obtained in Table 1 indicate that values of *σ*_1_ were comprised between 2.19 MPa and 6.58 MPa, which is in agreement with values found in literature, since typical values of bovine femoral trabecular bone strength when uniaxial stress is applied are comprised between 0.2 MPa and 16 MPa^40,44^.

Figure 6 illustrates that the evolution of the reflection coefficient of the BII as function of the compressive stress is significantly correlated to the mechanical behavior of the bone samples. As shown in Table 2, *R*_2_ and *R*_3_ were shown to decrease as a function of *σ* for *σ* < *σ*_2_ and for *σ* < *σ*_3_ respectively, with *σ*_2_ > *σ*_1_ and σ_3_ > *σ*_1_ for 94% of bone samples. For σ < *σ*_1_, which corresponds to ε < *ε*_*i*_ (see Fig. 2), bone tissue had an elastic behavior, so that the compression led to an elastic deformation of the trabecular network which was pressed onto the implant surface. However, local stresses near the BII may exceed the elastic limit and this compression in the macroscopic elastic regime may lead to an increase of the BIC, explaining the decrease of *R*_2_ and *R*_3_. Moreover, during this elastic compression (σ < *σ*_1_), the deformation of the trabecular network led to an increasing bone density, which also explains the decrease of *R*_2_ and *R*_3_ because it contributed to a decrease of the gap of acoustical properties at the BII.

Then for σ = *σ*_1_, which corresponds to *ε*_*i*_ < ε < *ε*_*f*_ (see Fig. 2), the trabeculae may fracture and the trabecular network progressively collapses^42,45^. Debris will progressively fill the pores of the trabecular structure, which also results in an increase of the BIC as well as of the mass density and apparent bone density of the trabecular sample, leading to a decrease of *R*_2_ and *R*_3_.

Eventually, for σ > *σ*_1_, which corresponds to ε > *ε*_*f*_ (see Fig. 2), the BIC ratio is close to 100% and could not increase anymore because all pores had already been filled. However, the strain increased faster as a function of the stress, so that the bone density and stiffness also increased due to nonlinear effects in bone tissue^45^, which contributed to a decrease of *R*_2_ and *R*_3_. As a consequence, *R*_2_ and *R*_3_ were globally decreasing functions of the stress, and the mean values of *λ*_*i,k*_ and *λ*_*f,k*_ were both negative. However, *λ*_*i,k*_ was always lower than *λ*_*f,k*_ (see Table 2), which indicates that for σ > *σ*_2_ (respectively σ > *σ*_3_)*, R*_2_ (respectively *R*_3_) had a lower dependence on σ compared to the elastic regime (σ < *σ*_1_). This last result may be explained by the fact that the increase of the BIC during compression had a higher influence on the ultrasonic response of the BII than the changes of bone mechanical properties.

Figure 7a shows that *σ*_1_ increases as a function of the apparent density *ρ*, which may be explained by the fact that the mechanical strength of the trabecular bone samples increased as a function of density. Note that such result is in good agreement with previous results obtained in the literature^42,45^. Moreover, Fig. 7b shows that *σ*_2_ and *σ*_3_ increased as a function of the apparent density *ρ*, which may be explained by the fact that *σ*_2_ and *σ*_3_ were positively correlated to *σ*_1_ (see Fig. 6).

This study has several limitations. First, the error related to the determination of the values of *σ* can be explained by two phenomena. The first one has been quantified by Table 2 and comes from the error made on the ultrasonic measurement for a given sample. The second one arises when different samples are considered and comes from the dispersion of the values of *R*_*0*,2_ and *R*_*0*,3_ (see Table 2), which have standard deviations of 9.10^−3^. This standard deviation may come from the variability of microstructural properties of the bone directly in contact with the implant, such as porosity or mineral density, which are difficult to estimate. Assuming a linear variation of *R*_*k*_ as a function of *σ* and given the values of *λ*_*i,2*_ (respectively of *λ*_*i,3*_), a decrease of 9.10^−3^ on the measured value of *R*_*2*_ (respectively of *R*_*3*_) would correspond to an increase of the stress applied to the BII of 1.9 MPa (respectively of 1.6 MPa). Therefore, it is difficult to determine the stress applied to the BII directly from the ultrasonic measurements. Second, errors on strain measurements were introduced by stress relaxation effects of bone tissue due to the time necessary to realize the ultrasonic measurements, which should be decreased in future studies. Third, the present study was performed using coin-shaped flat implants in order to work in a standardized environment where the stress distribution is approximately uniform on the BII, which has the advantage of allowing to identify the effect of the compressive stress on the ultrasonic response of the BII.

## Conclusion

This study quantifies the influence of compressive stresses applied to the BII on its ultrasonic response. A significant decrease of the reflection coefficient of the BII as a function of the compressive stress was obtained during the elastic compression of the trabecular bone samples and during the collapse of the trabecular network, with an average slope of −4.82 GPa^−1^. The results may be explained by an increase of the BIC when trabecular bone is compressed onto the implant as well as by changes of bone material properties. The influence of the compressive stress on the ultrasonic response of the BII is particularly important until a plateau stress corresponding to bone fracture is reached. Future works should focus on studying the effect of the implant surface roughness and on considering clinically used implants since their complex geometry may highly affect the stress distribution. Moreover, the effect of shear stresses applied to the BII should also be investigated.

## Data Availability

The datasets generated and analyzed during the current study are available from the corresponding author on reasonable request.
